# Airline Ranking Using Social Feedback and Adapted Fuzzy Belief TOPSIS

**DOI:** 10.3390/e27080879

**Published:** 2025-08-19

**Authors:** Ewa Roszkowska, Marzena Filipowicz-Chomko

**Affiliations:** Faculty of Computer Science, Bialystok University of Technology, Wiejska 45A, 15-351 Białystok, Poland; m.filipowicz@pb.edu.pl

**Keywords:** airlines ranking, entropy measure, e-commerce, fuzzy decision making, online consumer review, ordinal scale, synthetic measure, Tripadvisor

## Abstract

In the era of digital interconnectivity, user-generated reviews on platforms such as TripAdvisor have become a valuable source of social feedback, reflecting collective experiences and perceptions of airline services. However, aggregating such feedback presents several challenges: evaluations are typically expressed using linguistic ordinal scales, are subjective, often incomplete, and influenced by opinion dynamics within social networks. To effectively deal with these complexities and extract meaningful insights, this study proposes an information-driven decision-making framework that integrates Fuzzy Belief Structures with the TOPSIS method. To handle the uncertainty and imprecision of linguistic ratings, user opinions are modeled as fuzzy belief distributions over satisfaction levels. Rankings are then derived using TOPSIS by comparing each airline’s aggregated profile to ideal satisfaction benchmarks via a belief-based distance measure. This framework presents a novel solution for measuring synthetic satisfaction in complex social feedback systems, thereby contributing to the understanding of information flow, belief aggregation, and emergent order in digital opinion networks. The methodology is demonstrated using a real-world dataset of TripAdvisor airline reviews, providing a robust and interpretable benchmark for service quality. Moreover, this study applies Shannon entropy to classify and interpret the consistency of customer satisfaction ratings among Star Alliance airlines. The results confirm the stability of the Airline Satisfaction Index (ASI), with extremely high correlations among the five rankings generated using different fuzzy utility function models. The methodology reveals that airlines such as Singapore Airlines, ANA, EVA Air, and Air New Zealand consistently achieve high satisfaction scores across all fuzzy model configurations, highlighting their strong and stable performance regardless of model variation. These airlines also show both low entropy and high average scores, confirming their consistent excellence.

## 1. Introduction

In the digital era, social media and online review platforms, such as TripAdvisor, Google Reviews, and Yelp, have become essential infrastructures for capturing public sentiment, consumer preferences, and collective evaluations of services and products [1,2]. Unlike traditional surveys or static feedback forms, these platforms generate large-scale, dynamic, and user-driven data that reflect public perception in real time. Electronic word of mouth (e-WOM), as realized through e-commerce sites like Amazon and eBay, popular apps like Yelp, Nextdoor, TripAdvisor, Skytrax, and social media platforms like Instagram, Facebook, Twitter, TikTok, and YouTube, is recognized as an important source of information [3,4,5,6]. It plays a significant role in consumer decision-making. Online consumer reviews, which evolved from traditional word-of-mouth, have become a crucial factor in purchasing decisions due to their accessibility and influence in the digital era. Katyal and Sehgal [5] offer a comprehensive literature review and propose a conceptual framework that outlines how review, source, and platform credibility impact consumer behavior, highlighting areas for future research. Based on a review of existing literature, the study highlighted three main factors—the credibility of the review, the reviewer, and the platform—which together shape consumer trust and influence purchasing decisions.

Platforms like TripAdvisor do not merely collect individual experiences but function as socially driven ecosystems where opinions evolve, spread, and influence others. Filieri et al. [6] noted that TripAdvisor’s number of monthly visitors grew “from 20 million in 2010 to 463 million in 2019”. These environments can be modeled as complex adaptive systems or networks, with users and service entities (e.g., airlines) as nodes, and their interactions, shared preferences, or evaluative similarities forming dynamic connections. Such networked structures capture the complex informational dynamics of modern society, where individual feedback contributes to emergent patterns of satisfaction, trust, or dissatisfaction.

Airline service quality has been extensively studied using a range of methodological approaches. Classic models such as SERVQUAL (Service Quality) and SERVPERF (Service Performance) continue to be widely applied to assess customer expectations and perceptions, revealing important service dimensions and cultural influences on passenger evaluations [7,8]. However, these approaches often fall short in managing the ambiguity and complexity of subjective ratings. To address these limitations, multi-criteria decision-making (MCDM) techniques have been proposed to capture data structure. Methods like fuzzy AHP (Analytic Hierarchy Process), TOPSIS (Technique for Order Preference by Similarity to Ideal Solution), VIKOR (VIšekriterijumska optimizacija i kompromisno rangiranje), and MUSA (MUlticriteria Satisfaction Analysis) enable structured prioritization and ranking of airlines across multiple service attributes, accounting for the imprecision inherent in user feedback [9,10,11,12]. Additionally, data-driven techniques such as machine learning and text mining complement these methods by extracting sentiment and thematic insights from large-scale unstructured review data [13,14]. Statistical and behavioral models further provide a macro-level understanding of systemic biases and rating patterns shaped by cultural contexts [15,16].

The data often appear in unstructured formats such as free-text comments, star ratings, or images, while ordinal scale responses provide a rich source of consumer experiences, particularly in service-oriented sectors like airlines, hotels, hospitality, and food services. Airline reviews provide a clear example of this phenomenon. Passengers frequently evaluate multiple service aspects, including punctuality, travel comfort, customer support, boarding procedures, and in-flight meals using linguistic ordinal scales like “Poor,” “Average,” or “Excellent.” These ratings inherently express subjective, vague, and sometimes inconsistent perceptions. This linguistic vagueness and ambiguity pose significant challenges for computational aggregation and meaningful analysis, especially when aiming to produce reliable and interpretable service quality rankings.

Summing up, in many online review settings, a single evaluation question, such as an overall satisfaction score, is often rated by respondents using ordinal-scale linguistic categories (e.g., Poor, Fair, Good, Excellent). The core challenge lies in aggregating these responses in a way that preserves their semantic meaning. Traditional approaches based on the simple arithmetic mean of ordinal ratings risk distorting these meanings and overlooking the inherent uncertainty in such evaluations. Our approach addresses this challenge through three key contributions, as follows:

- **Semantic preservation of linguistic categories**, ensuring that the meaning of evaluation terms is retained and avoiding distortions introduced by applying arithmetic means to ordinal data.
- **Stability analysis across multiple fuzzy utility functions and linguistic scales**, by applying alternative fuzzy set representations to examine how ranking results change or remain stable under varying assumptions.
- **Integration with entropy analysis** to assess rating consistency and connect stability patterns with performance rankings.

This framework not only processes large-scale data efficiently but also models uncertainty in a way that reflects the complexity and dynamics of modern opinion networks.

Building on this rich methodological landscape and addressing challenges with linguistic online reviews, this study proposes a novel hybrid framework that integrates the Fuzzy Belief Structure (FBS) model with the TOPSIS method. The Airline Satisfaction Index (ASI) is based on the TOPSIS with Belief Structure approach [17] and the BS-TOSIE (Belief Structure-Based TOPSIS for Survey Item Evaluation) method [18], extending the latter to fuzzy environments by incorporating Fuzzy Belief Structure-based TOPSIS from [19]. This extension is specifically designed to analyze single-question, linguistically expressed online feedback.

The FBS model enables the representation of subjective user ratings as fuzzy belief distributions over satisfaction levels, capturing uncertainty and partial belief inherent in linguistic assessments. These belief profiles are aggregated and compared to ideal and anti-ideal service benchmarks using a Belief Distance Measure, allowing the construction of a composite ranking that reflects the relative closeness of each airline to an optimal satisfaction profile.

The proposed ASI is designed to evaluate and rank airlines based on online user reviews, effectively preserving the subjectivity and imprecision inherent in linguistic ratings while enabling rigorous, data-driven analysis. Applied to a real-world dataset of TripAdvisor airline reviews, the method demonstrates its capability to handle large-scale, imprecise, and linguistically rich user-generated content.

We evaluate customer satisfaction for 25 international Star Alliance airlines using a hybrid analytical framework that combines fuzzy logic modeling with multi-criteria evaluation. This approach captures the uncertainty inherent in subjective customer feedback, especially from sources like TripAdvisor, where reviews are often unstructured or incomplete. Our analysis proceeds in two stages, as follows:

1. Five versions of the Airline Satisfaction Index (ASI1−ASI5) are constructed using different fuzzy utility functions to model linguistic ratings and assess how variations in utility assumptions affect airline rankings. These results are then compared with the overall rating provided by the TripAdvisor platform, which is based on the arithmetic mean of individual review scores.
2. Integration of ratings across nine key service dimensions to complement fuzzy satisfaction scores, providing a detailed view of airline performance.

This methodology addresses the following research questions:

- RQ1: *How robust are Star Alliance airlines’ satisfaction rankings when different shapes of fuzzy utility functions are applied?*
- RQ2: *Which Star Alliance airlines consistently achieve high satisfaction across varying fuzzy models?*
- RQ3: *How do the different Airline Satisfaction Indexes compare with the average rating calculated using the arithmetic mean?*
- RQ4: *What specific categories of Star Alliance airlines’ service satisfaction reveal underlying performance gaps when assessed through TripAdvisor data?*

Additionally, this research aims to examine how Shannon entropy [20,21] can be applied to detect inconsistencies in passenger satisfaction data and support strategic recommendations for service quality improvement across global airline alliances. Shannon entropy, introduced by Claude Shannon in 1948 [20], is a foundational concept in information theory that quantifies the uncertainty or randomness associated with a probability distribution. It measures the expected amount of information generated by a stochastic process and has since found wide application in various disciplines, including economics, finance, and management [22,23,24], data analysis, and machine learning [25] due to its effectiveness in capturing variability and systemic disorder. Paper [26] presents a concise overview of key entropy measures, their evolution, and broad interdisciplinary applications in quantifying uncertainty across domains such as finance, AI, and thermodynamics. In the context of passenger satisfaction ratings, Shannon entropy serves as a valuable tool for evaluating the consistency or diversity of customer opinions about specific airlines. Specifically, entropy is used to quantify the variability of passenger ratings across five qualitative categories: Excellent, Good, Average, Poor, and Terrible. A low entropy value indicates that feedback is concentrated within a limited range of categories—such as predominantly positive or predominantly negative responses—suggesting a relatively uniform perception among passengers. In contrast, a high entropy value reflects a more even distribution of ratings, indicating greater uncertainty or divergence of opinion. This analytical perspective leads to the following complementary research question:

- RQ5: *How can Shannon entropy be utilized to identify inconsistencies in customer satisfaction ratings among Star Alliance airlines, and is the Shannon entropy measure consistent with the* ASI *indexes and the average TripAdvisor rating?*

This study contributes to the literature by advancing the modeling of opinion dynamics within social evaluation systems and developing an information-based framework for analyzing user feedback. It emphasizes the network-driven emergence of collective satisfaction and dissatisfaction patterns and introduces a synthetic indicator construction method tailored to complex social systems characterized by linguistic uncertainty and decentralized data generation. By transforming vague and inconsistent online feedback into meaningful service quality evaluations, ASI bridges subjective linguistic input with quantitative analysis, thereby enhancing decision-making in dynamic, user-driven contexts.

The paper is structured as follows. Section 2 provides a literature review of existing approaches to airline service quality evaluation. Section 3 describes the problem, presents the data source, and introduces the proposed ASI methodology. Section 4 discusses the results of applying the ASI to rank airlines based on TripAdvisor reviews, followed by an interpretation of the findings. Finally, the paper concludes with a summary of key insights and suggestions for future research.

## 2. Methodological Approaches in Airline Service Quality Research

Airline service quality has been extensively investigated using diverse methodological approaches, revealing a broad spectrum of priorities, passenger expectations, and evaluation mechanisms. The reviewed studies can be thematically grouped based on the methodological approach employed in evaluating service quality: traditional SERVQUAL and SERVPERF-based models, MCDM techniques, machine learning and text mining approaches, as well as advanced statistical modeling such as structural equation modeling (SEM).

A considerable number of studies utilize the SERVQUAL or SERVPERF frameworks—either in their original or modified versions—to evaluate customer expectations and perceptions. Pakdil and Aydın [7] analyzed weighted SERVQUAL scores using factor analysis, revealing that responsiveness was the most valued dimension, while availability ranked lowest. Their study also highlighted demographic factors (such as education) that influence the gaps between expectations and perceptions. Basfirinci and Mitra [8] provided a more nuanced view by integrating SERVQUAL with the Kano model to investigate cross-cultural differences in customer perceptions between Turkey and the USA. While gap scores were negative in both countries, the importance of specific attributes varied by cultural context, suggesting the need for both global and localized service customization. Similarly, Saha and Theingi [27] studied low-cost carriers using a modified SERVPERF and structural equation modeling (SEM) to explore the relationships between service quality, satisfaction, and behavioral intentions. Their findings indicated that flight schedules were the most critical factor influencing passenger satisfaction and loyalty.

Another group of studies utilizes MCDM techniques to structure service quality evaluations and benchmark airline performance. Tsaur et al. [9] applied fuzzy set theory in combination with AHP and TOPSIS to handle the inherent subjectivity in service evaluations, identifying courtesy, safety, and comfort as the most critical attributes, while empathy received the least attention. Gupta [10] employed a hybrid MCDM framework combining the Best-Worst Method (BWM) and VIKOR to prioritize service quality attributes and rank Indian airlines. Tangibility, reliability, safety, and ticket pricing emerged as the most important factors, with VIKOR revealing performance differences among five airlines. Tsafarakis et al. [11] used MUSA to assess passenger satisfaction for Aegean Airlines, enabling managers to target service elements that scored high in importance but low in performance. Roszkowska et al. [12] proposed a Synthetic Measure for Ordinal Data (SMOD), based on Hellwig’s method, to rank airlines based on online multi-criteria review data. Singapore Airlines was ranked highest among Star Alliance carriers.

A third category of research focuses on computational, data-driven techniques such as text mining, sentiment analysis, and synthetic evaluation. These represent the latest advancements in airline service quality analysis. Sezgen et al. [13] applied Latent Semantic Analysis to over 5000 TripAdvisor reviews, showing how satisfaction drivers differ by carrier type (low-cost vs. full-service) and travel class (economy vs. premium). Common dissatisfaction drivers included seat comfort, disruptions, and staff behavior. Chang et al. [14] employed deep learning and aspect-based sentiment analysis using BERT models on TripAdvisor reviews, particularly examining sentiment shifts during COVID-19. Their results demonstrated that transformer-based models outperform traditional sentiment classifiers and provide deeper insights into passenger concerns. Lucini et al. [28] used text mining and LDA on over 55,000 online reviews to identify key satisfaction dimensions influencing airline recommendations, highlighting the importance of cabin staff, onboard service, and value for money across different passenger classes. Hwang et al. [29] applied machine learning to customer feedback and satisfaction ratings, achieving over 83% accuracy in predicting airline customers’ return visits, with longer comments improving prediction quality. Korfiatis et al. [30] used Structural Topic Models to analyze airline reviews, showing that combining text and ratings better explains customer satisfaction and highlights factors behind low-cost carriers’ market success. Kwon et al. [31] applied topic modeling and sentiment analysis on 14,000+ online reviews of Asian airlines, identifying delays as a major dissatisfaction factor and staff service as a key driver of customer satisfaction.

Lastly, studies grounded in statistical and behavioral analyses offer macro-level insights into consumer behavior. Gursoy et al. [15] used correspondence analysis to evaluate the relative market positioning of 10 U.S. airlines based on the Department of Transportation (DOT) metrics, offering valuable strategic insights. Stamolampros et al. [16] examined domestic bias in customer ratings using Hofstede’s cultural dimensions, finding that cultural orientation influences rating patterns. These behavioral models help uncover systemic biases and preferences in service evaluations. Ban and Kim [32] used semantic network analysis (CONCOR) and linear regression on 9632 online reviews to examine airline customer satisfaction. All factors except entertainment had a significant impact on satisfaction and recommendation.

Taken together, this selected review highlights the rich methodological diversity in airline service quality research. Table 1 synthesizes the studies by analytical method, focus, and key findings.

In conclusion, this brief literature review demonstrates that no single methodology can fully capture the complex nature of airline service quality. Traditional SERVQUAL-based instruments remain effective for identifying perceptual gaps, while MCDM techniques provide valuable support for managerial decision-making. At the same time, AI-driven models open new avenues for analyzing user-generated content, and statistical frameworks enhance the understanding of broader systemic patterns.

However, each approach has inherent limitations that should be considered. SERVQUAL-based instruments may not fully capture latent patterns in large datasets and can be influenced by cultural differences or unstructured feedback. MCDM techniques depend on subjective weight assignments and the assumptions underlying aggregation methods, which can affect ranking outcomes. AI-driven and text-mining models require large, high-quality datasets and can be sensitive to noise or bias, while their complexity may limit interpretability. Statistical and behavioral models, although useful for uncovering macro-level patterns, often rely on aggregated or structured data, potentially overlooking individual preferences and being affected by cultural or systemic biases. The complementary application of these methods offers a more comprehensive understanding of passenger satisfaction and supports the development of tailored quality improvement strategies, while acknowledging these limitations ensures a balanced and robust perspective for future research.

## 3. Materials and Methods

### 3.1. Problem Description and Data Source

Star Alliance, founded in 1997 by five airlines, is the world’s first global airline alliance. Today, it includes 25 members offering seamless travel through over 50 hubs. Its management, based in Frankfurt and Singapore, coordinates joint services to enhance the passenger experience [33].

**Figure 1:**
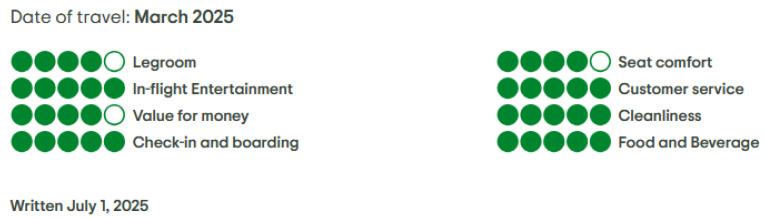
Individual criteria-based ratings for Singapore Airlines from TripAdvisor [34] (accessed on 12 July 2025).

On the TripAdvisor platform, passengers rate their airline experience based on nine specific criteria: C1–Legroom, C2–Seat comfort, C3–In-flight entertainment (WiFi, TV, movies), C4–Onboard experience, C5–Customer service, C6–Value for money, C7–Cleanliness, C8–Check-in and boarding, and C9–Food and beverage. These aspects are assessed using a graphical interface where users mark the proper number of circles to reflect their opinion. Each circle corresponds to a verbal label: 1–Terrible, 2–Poor, 3–Average, 4–Good, and 5–Excellent (see Figure 1).

**Figure 2:**
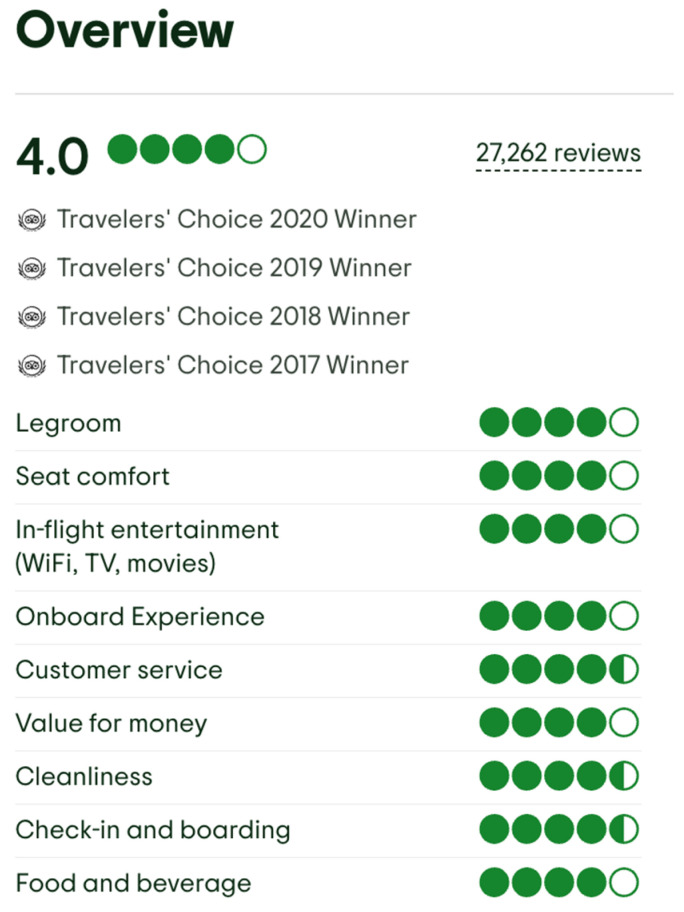
Aggregated area ratings for Singapore Airlines from TripAdvisor [34] (accessed on 12 July 2025).

In addition to individual ratings and written reviews, TripAdvisor also provides an overall satisfaction score for each airline, along with the distribution of user responses across a five-point scale. Figure 2 displays a sample of the aggregated area ratings for Singapore Airlines, as retrieved from the TripAdvisor website.

**Figure 3:**
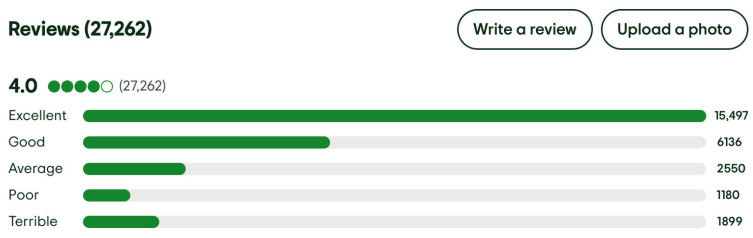
The distribution of user responses for overall satisfaction across the five-point scale for Singapore Airlines from TripAdvisor [34] (accessed on 12 July 2025).

Figure 3 presents the distribution of user responses for overall satisfaction across the five-point scale.

Table 2 summarizes the distribution of overall satisfaction ratings and average scores for airlines belonging to the Star Alliance. Additionally, Shannon entropy is calculated to measure the variability of customer satisfaction ratings across an ordinal five-point scale: Excellent, Good, Average, Poor, and Terrible. These values are presented in the last column of Table 2. Entropy quantifies the degree of uncertainty or inconsistency in how passengers evaluate a given airline. The formula is defined as follows [20]:

(1) $$H=\;-\;\sum_{i=1}^{n}p_{i}\log_{2}\left(p_{i}\right),$$

where H is the entropy value representing the dispersion of customer opinions for a specific airline; n is the number of rating categories (in this case, n = 5); $p_{i}$ is the relative frequency (i.e., empirical probability) of ratings falling into the i-th category for a given airline.

The base-2 logarithm ensures the result is expressed in bits, representing the average information content. A low entropy value indicates that most ratings are concentrated in one or two categories, reflecting a consistent perception of service quality among passengers. In contrast, a high entropy value suggests that ratings are more evenly distributed across all categories, implying divergent opinions or inconsistent service experiences. This measure helps compare airlines not only based on average satisfaction, but also in terms of the stability and coherence of customer feedback.

**Figure 4:**
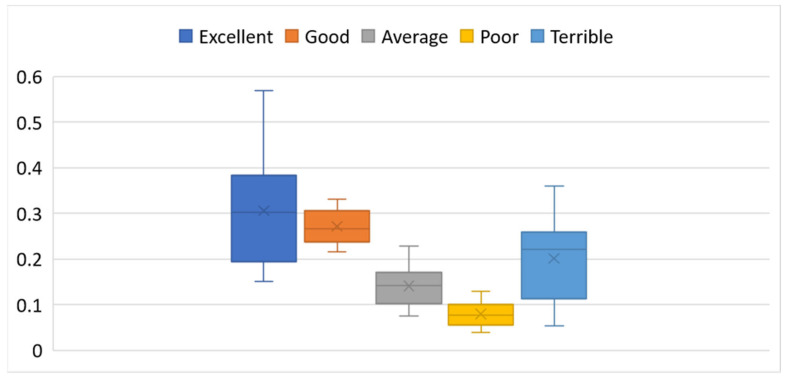
Box plots for the frequencies of ratings on a 5-point ordinal scale.

Descriptive statistics for the distribution of linguistic ratings are presented in Table 3, while their spread and variability are visualized through box plots in Figure 4.

This analysis examines customer satisfaction across 25 global airlines using relative frequencies of ratings on a 5-point ordinal scale: Excellent, Good, Average, Poor, and Terrible. The data reflect the proportion of responses each airline received in these categories, offering a standardized comparison of perceived service quality (Table 2, Figure 4).

The “Excellent” category is characterized by a variability of 41.6%, with an average frequency of approximately 30.6%, but ranging widely from 15.1% for Air China to a remarkable 56.8% for Singapore Airlines. Other top-rated airlines in this category include Air New Zealand (55.0%), EVA Air (51.0%), and ANA (All Nippon Airways) (50.6%). These airlines stand out as industry leaders, consistently delivering superior service that earns them the highest customer praise. In contrast, Air India, Shenzhen Airlines, and TAP Air Portugal all fall below 19%, reflecting limited recognition for excellence.

The “Good” category is quite stable, with relatively low dispersion (standard deviation 0.037). On average, 27.2% of customers rated their experiences in this category. Airlines such as Thai Airways (33.1%), Shenzhen Airlines (32.9%), and Asiana Airlines (32.7%) perform well here, suggesting strong but not necessarily outstanding service. Notably, Singapore Airlines and Air New Zealand, despite receiving outstanding “Excellent” ratings, exhibit lower variability, indicating a strong customer tendency to assign them the highest possible ratings rather than moderate ones.

The “Average” category offers insight into passenger indecision or neutrality. The mean frequency is 14.1%; however, Shenzhen Airlines (22.8%) and Air China (22.3%) exhibit significantly higher scores, suggesting these airlines evoke a mixed or moderate response. Conversely, AEGEAN Airlines (7.4%) and EVA Air (8.0%) receive fewer “Average” ratings, indicating more polarized customer perceptions, either positive or negative.

In the “Poor” category, while the average is relatively low (7.9%), it remains an important indicator of weak service areas. Air China, United Airlines, and Air Canada each exceed 11% in this category, pointing to notable dissatisfaction among their passengers. By contrast, EVA Air, ANA, AEGEAN, and Singapore Airlines each have less than 4.5% of passengers rating them as “Poor,” reinforcing their reputations for consistently high service standards.

The most revealing is the “Terrible” category, which has the highest variability of all. On average, 20.2% of passengers gave the lowest possible score, but the frequency ranges from just 5.4% for ANA to an alarming 36.0% for Air India. Other carriers with high rates of “Terrible” ratings include TAP Air Portugal, LOT Polish Airlines, and Air Canada, all of which exceed 29%. These figures highlight significant performance issues, likely tied to service inconsistencies, delays, or poor customer support. On the other end of the spectrum, Singapore Airlines, EVA Air, and Air New Zealand maintain low “Terrible” rates, further validating their positive reputations.

A comparison of these five rating categories reveals clear patterns, as follows:

- Airlines such as Singapore Airlines, ANA, Air New Zealand, and EVA Air consistently rank in the top tiers and receive minimal negative feedback, indicating highly reliable service experiences.
- Mid-tier performers such as Asiana Airlines, Shenzhen Airlines, and Thai Airways receive consistently “Good” and “Average” scores, pointing to satisfactory but not exceptional experiences.
- Carriers such as Air India, LOT Polish Airlines, and Air Canada record a high proportion of “Poor” and “Terrible” ratings, highlighting significant service-related issues.

Entropy Airlines’ values were grouped into four classes based on the following quartiles:

- Class I: Very Low Entropy [Min; Q1)—Highly consistent feedback.
- Class II: Low Entropy [Q1; Me)—Generally consistent feedback with minor variance.
- Class III: Moderate Entropy [Me; Q3)—Noticeable variability in customer feedback.
- Class IV: High Entropy [Q3; Max]—Strong disagreement and service inconsistency.

**Figure 5:**
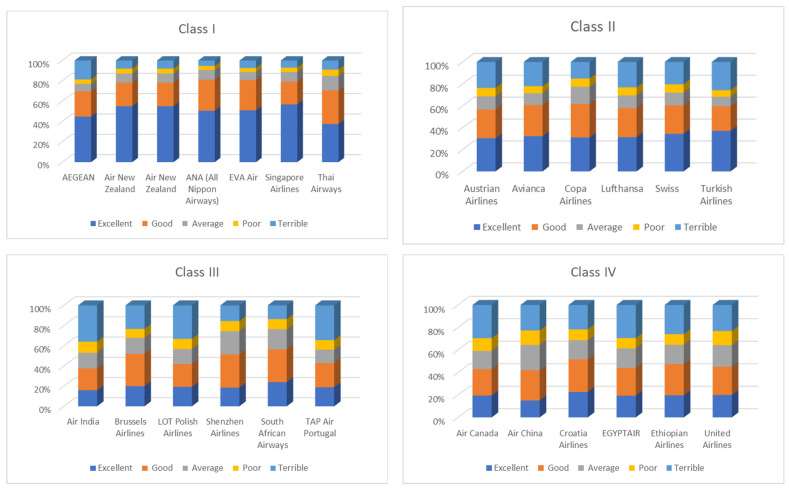
Distribution of customer ratings across entropy classes (I–IV) based on Tripadvisor data [35] (accessed on 12 July 2025).

Distribution of customer ratings across entropy classes is presented in Figure 5.

Table 4 presents a simplified yet comprehensive view of how airlines are grouped based on the entropy of customer satisfaction ratings. Entropy is used here as a statistical indicator of consistency in customer experience. A lower entropy value reflects uniform, predictable feedback, while a higher entropy indicates fragmentation, polarization, or inconsistency. This classification helps identify service performance trends and highlights strategic implications for customer experience management. Refer to Figure 5 for the visual distribution of ratings across classes.

Table 5 provides ordinal ratings for 25 Star Alliance member airlines across nine service-related criteria, from Legroom (C1) to Food and beverage quality (C9). The ratings are given on a five-point scale with 0.5-point intervals, where higher values reflect higher levels of passenger satisfaction.

**Figure 6:**
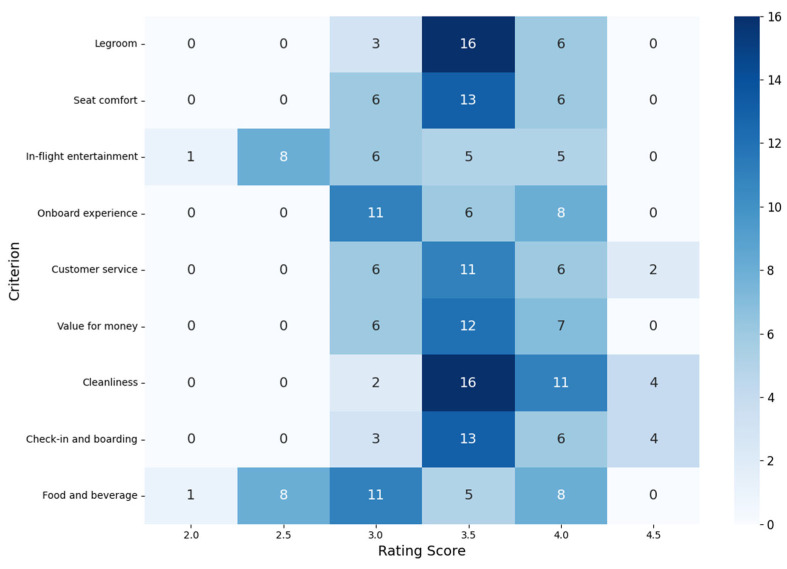
Heatmap for different aspects of airline service quality based on Tripadvisor data [35] (accessed on 12 July 2025).

As shown in Figure 6, the heatmap visualizes how frequently different aspects of Star Alliance airlines’ service quality were rated on TripAdvisor, based on the data presented in Table 5.

While the scores in Table 5 suggest differences in performance, it is important to emphasize that they are ordinal. Therefore, instead of interpreting them as precise numerical values, we focus on the distribution of ratings to identify meaningful patterns in perceived service quality.

A clear performance trend emerges (see Table 5). Some airlines consistently receive high ratings across nearly all criteria, suggesting strong, broad-based passenger approval. Airlines such as Singapore Airlines, Air New Zealand, EVA Air, and ANA (All Nippon Airways) stand out for achieving ratings of 4.0 or above in almost every category. Notably, Singapore Airlines and Air New Zealand receive top marks in Customer service, Cleanliness, and Check-in and boarding, with ratings reaching 4.5, which aligns with their global reputations for high-quality service and customer satisfaction.

In contrast, airlines such as Air India, TAP Air Portugal, Air China, and Croatia Airlines tend to score lower, often falling in the 2.5–3.0 range in categories like In-flight entertainment, Seat comfort, Onboard experience, Customer service, and Food and beverage. These results may reflect either customer dissatisfaction in key service categories or business models that prioritize basic service delivery over enhanced passenger experience.

A large number of Star Alliance members occupy a middle ground. Their ratings typically fall within the 3.0 to 3.5 range across most criteria, reflecting consistent but unexceptional service quality. These airlines may appeal to a broad range of travelers, offering reliability without standing out in any particular category. Among them, Air Canada, Ethiopian Airlines, and United Airlines stand out for their uniformity as all of their ratings, including the overall score, fall strictly within the 3.0 to 3.5 range with no deviations. In addition, several other carriers, such as Austrian Airlines, South African Airways, Lufthansa, and Avianca, also fall predominantly within this mid-range, with just a single exception; each of them received a 4.0 rating in the seat comfort category C7, suggesting a modest strength in that particular area while maintaining otherwise average performance.

To deepen the analysis, a frequency distribution of scores across all criteria further highlights key trends (see Figure 6). The most frequently occurring rating is 3.5, which dominates in areas such as Cleanliness and Legroom, suggesting that many airlines are perceived as adequate to good in these core operational aspects. For instance, Cleanliness alone received 16 ratings of 3.5—the highest count for any score in the dataset, along with eleven ratings of 4.0. In contrast, Food and beverage, as well as In-flight entertainment, emerge as relatively weak points across the alliance. Both categories exhibit concentrated clusters of lower ratings, with eight ratings of 2.5 and eleven ratings of 3.0 in the former, and six ratings of 3.0 in the latter. These patterns point to uneven quality or limited offerings in these areas, even among airlines that otherwise perform well. Only a few airlines receive ratings above 4.0 in these dimensions.

In contrast, Value for money stands out with a relatively strong distribution: it received twelve ratings of 3.5, seven of 4.0, and six of 3.0, indicating that passengers often feel they receive appropriate service for the price, particularly with top-performing carriers. Similarly, Customer service shows a favorable distribution, with the majority of scores at 3.5 or higher, further reinforcing the positive perception of airlines such as Air New Zealand and Singapore Airlines. Notably, ratings of 2.0 are rare, occurring only once each in the categories of In-flight entertainment and Food and beverage. This suggests that severe dissatisfaction is uncommon, and most airlines maintain at least a baseline level of acceptable service.

In conclusion, the patterns of ordinal ratings from TripAdvisor offer a nuanced perspective on passenger experiences across Star Alliance airlines and service categories. While top-tier carriers such as Singapore Airlines, Air New Zealand, EVA Air, and ANA consistently achieve high ratings across the board, other airlines show room for improvement, particularly in areas like in-flight entertainment and catering. By analyzing the distribution of ordinal scores rather than interpreting them as interval data, we gain a more accurate understanding of customer sentiment and service consistency within the alliance. These insights can support strategic improvements and help set more realistic expectations for travelers choosing among Star Alliance carriers.

### 3.2. Foundations and Motivation for Using Fuzzy Belief Structure with TOPSIS

The foundational Belief Structure (BS) model was originally developed in [36,37,38] and later extended through the incorporation of fuzzy numbers, resulting in the Fuzzy Belief Structure (FBS) representation [19,39]. These approaches have been integrated into hybrid multi-criteria decision-making (MCDM) methods, including TOPSIS [17,18,19,39] or VIKOR [40,41]. The application of BS and FBS in MCDM has proven particularly valuable in domains where human judgment and linguistic evaluations play a central role. Key areas of application include risk assessment [39], urban delays [41], hybrid efficiency evaluation [40], and quality of life analysis [42].

To evaluate respondents’ feedback on individual online questions related to airline services, particularly on open review platforms such as TripAdvisor, Google Reviews, and various social media, we propose the Airline Satisfaction Index (ASI), a hybrid approach grounded in the methodologies presented in [17,18,19]. The ASI effectively manages subjective ordinal responses by combining the Fuzzy Belief Structure (FBS) with the TOPSIS method. This integration enables structured aggregation, comparison, and ranking while preserving the inherent uncertainty and vagueness of linguistic data. Unlike traditional statistical techniques such as computing arithmetic means of ordinal ratings, ASI maintains the semantic integrity of linguistic scales, offering a more accurate reflection of user perceptions, especially when feedback is imprecise or ambiguous, as is common on social media.

Addressing the challenges of linguistic vagueness in user-generated evaluations ASI avoids averaging ordinal data, which can distort meaning, and instead captures the distribution of belief across fuzzy linguistic categories. This results in a robust and interpretable model for assessing perceived satisfaction, making ASI particularly suitable for analyzing single-question evaluations from diverse respondents, including reviews of airlines, hotels, or other services.

The following subsection outlines the detailed steps of the ASI method.

### 3.3. Fuzzy Belief Structure TOPSIS for Online Satisfaction: Airline Satisfaction Index (ASI)

The following section presents the step-by-step procedure of the proposed Airline Satisfaction Index (ASI), designed to systematically evaluate and rank online satisfaction assessments based on online linguistic user reviews.

Step 1. Data Collection

User-generated online responses are collected and categorized using an ordinal linguistic scale (e.g., Poor, Average, Excellent). Let the set of evaluated objects (e.g., services, products) be $O=\left\{O_{1},\;O_{2},\ldots ,O_{m}\right\}$ each evaluated according to N linguistic categories, later referred to as grades, $H=\left\{H_{1},H_{2},\ldots ,H_{N}\right\}$ with $H_{1}$ representing the most favorable grade and $H_{N}$ the least favorable. Non-responses may be recorded as a separate category or excluded. In this procedure, we assume full responses. As the analysis is based on a single question concerning the overall satisfaction score, the dataset contains no missing reviews.

For each object $O_{i}$, let $n_{i}$, denote the total number of respondents who evaluated it. For each grade $H_{k}$, let $n_{ik}$ represent the number of respondents who assigned a grade $H_{k}$ to the object $O_{i}$.

Step 2. Determine the Fuzzy Utility Function and Similarity Matrix

To account for the imprecise and linguistic nature of user-generated evaluations, each linguistic grade $H_{k}$ is modeled as a fuzzy number over a normalized satisfaction scale [0, 1]. Depending on the desired level of granularity, these fuzzy numbers can take either triangular or trapezoidal forms.

Utility values are assigned to evaluation grades and used to calculate the similarity between them to measure their closeness.

Let

(2) $$U\left(H_{k}\right)=\left(u_{1}^{k},u_{2}^{k},u_{3}^{k}\right)$$

for triangular fuzzy numbers, k=1, 2,…,N,

and

(3) $$\;U(H_{k})=(u_{1}^{k},u_{2}^{k},u_{3}^{k},u_{4}^{k})$$

for trapezoidal fuzzy numbers, k=1, 2,…,N.

The similarity between grades $H_{i}$ and$\;H_{j}$ is calculated as follows:

(4) $${\tilde{s}}_{ij}\left(H_{i},H_{j}\right)=1-\sum_{s=1}^{3}\frac{\left|\;u_{s}^{i}-u_{s}^{j}\right|}{3}\;$$

for triangular fuzzy numbers, i,j=1,2,…,N, and

(5) $${\tilde{s}}_{ij}\left(H_{i},H_{j}\right)=1-\sum_{s=1}^{4}\frac{\left|\;u_{s}^{i}-u_{s}^{j}\right|}{4}.\;$$

for trapezoidal fuzzy numbers, i,j=1,2,…,N.

These similarity values form the matrix

(6) $$\tilde{S}=\left[{\tilde{s}}_{ij}\right],$$

capturing the degree of closeness between evaluation categories.

Step 3. Construction of Fuzzy Belief Structures

Building on the fuzzy modeling of evaluation grades $H_{k}$ introduced in Step 2, we now define the Fuzzy Belief Structure (FBS) for each evaluated object $O_{i}$. The FBS captures the distribution of subjective assessments provided by respondents in terms of belief degrees assigned to each fuzzy linguistic category.

For each object, compute the fuzzy belief degree $\beta_{ik}$ for each object $O_{i}$ and grade $H_{k}$ as follows:

(7) $$\beta_{ik}=\frac{n_{ik}}{n_{i}}$$

where $n_{i}=\sum_{k=1}^{N}n_{ik}$ is the total number of respondents evaluating $O_{i}$, $n_{ik}$—the number of respondents who assigned a grade $H_{k}$ to the object $O_{i}.$

The Fuzzy Belief Structure (FBS) for $O_{i}$ is defined as the set of belief degrees assigned to each grade

(8) $$FBS\left(O_{i}\right)=\left\{\left(H_{k};\;\beta_{ik}\right);\;k=1,\ldots ,N\right\}$$

or equivalently as a belief vector

(9) $$S_{i}=\left[\;\beta_{i1},\;\beta_{i2},\ldots \beta_{iN}\right]$$

satisfying $\sum_{k=1}^{N}\beta_{ik}=1$, where $\beta_{ik}\in \;\left[0,1\right].$ Each $\beta_{ik}$ represents the proportion of respondents who assigned the fuzzy linguistic rating${\;H}_{k}$ to object $O_{i}$, thereby reflecting the strength of belief that $O_{i}$, belongs to category ${\;H}_{k}$. This structure enables a faithful representation of subjective evaluations under uncertainty, while preserving the semantics of the fuzzy linguistic scale established in Step 2.

Step 4. Identify Ideal and Negative-Ideal Belief Solutions

Define reference vectors representing the best and worst possible evaluations.

The Positive Ideal Belief Solution (PIBS) represents the best possible evaluation outcome;

(10) $$A^{+}=\left[1,\;0,\ldots ,0\right]$$

The Negative Ideal Belief Solution (NIBS) represents the worst-case scenario, as follows:

(11) $$A^{-}=\left[0,\ldots ,0,\;1\right].$$

Step 5. Compute Separation Measures

To assess how close each object is to the ideal and worst cases, compute separation measures using a belief-based distance metric, where comparison between two FBS models is transformed into a distance between two vectors. The distance from PIBS for object $O_{i}\;$ is the following:

(12) $$D^{+}\left(A_{i}\right)=D_{i}^{+}=d_{BS}\left(\;S_{i},A^{+}\right)={\left(\frac{1}{2}\left(\;S_{i}-A^{+}\right)\tilde{S}{\left(\;S_{i}-A^{+}\right)}^{T}\right)}^{\frac{1}{2}}$$

where i=1, 2,…,m.

Similarly, the distance from NIBS for the object $O_{i}$ is the following:

(13) $$D^{-}\left(A_{i}\right)=D_{i}^{-}=d_{BS}\left(\;S_{i},A^{-}\right)={\left(\frac{1}{2}\left(\;S_{i}-A^{-}\right)\tilde{S}{\left(\;S_{i}-A^{-}\right)}^{T}\right)}^{\frac{1}{2}}$$

where i=1, 2,…,m.

Step 6. Calculate the Relative Closeness

Compute the relative closeness of each object $O_{i}$ to the ideal solution

(14) $$ASI\left(O_{i}\right)=\frac{D_{i}^{-}}{D_{i}^{-}+D_{i}^{+}}\;.$$

where i=1,2,…,m.

A higher $ASI\left(O_{i}\right)$ indicates greater similarity to the ideal outcome and thus higher perceived satisfaction.

Step 7. Rank the Objects

Based on the values of$\;ASI\left(O_{i}\right)$, rank the objects from most to least favorable. The object with the highest relative closeness$\;ASI\left(O_{i}\right)\;$is considered the best-performing in terms of perceived satisfaction among respondents.

**Figure 7:**
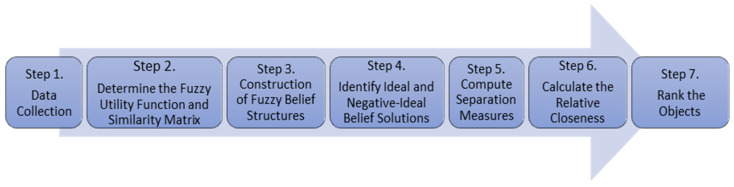
Schema of the proposed ASI approach, illustrating the systematic steps of evaluation and ranking.

The proposed ASI approach consists of several systematic steps that guide the evaluation and ranking process (see Figure 7).

### 3.4. Capturing Subjective Evaluations: Fuzzy Modeling of TripAdvisor Review Scales and Fuzzy Belief Structure

Fuzzy logic provides a powerful framework for capturing subjective and linguistically expressed concepts such as satisfaction, which are inherently vague and ambiguous. In fuzzy modeling, these linguistic variables are represented by fuzzy sets, typically using triangular or trapezoidal fuzzy numbers. This allows for a flexible representation of the gradual and imprecise nature of human evaluations [43,44,45].

This approach aligns with broader trends in fuzzy decision-making research, which emphasize the need for models capable of processing unstructured, real-world human feedback [46,47]. It has been widely applied in decision-making contexts where natural language is preferred over precise numerical input. Herrere-Viedma et al. [46] in comprehensive reviews of fuzzy and linguistic decision-making over the past five decades highlighted that fuzzy logic enables the modeling of decision problems characterized by uncertainty, imprecision, and diverse human perceptions. The integration into multi-criteria, group, and crowd-based decision frameworks has made fuzzy logic a valuable tool for modern applications that involve collective and subjective judgments.

One such application is the analysis of user-generated content on platforms like TripAdvisor, where feedback is expressed using a 5-point ordinal scale: Excellent, Good, Average, Poor, and Terrible. Within the Fuzzy Belief Structure (FBS) framework, these expressions are treated as grades: $H_{1}$: Excellent; $H_{2}$: Good; $H_{3}$: Average;$\;H_{4}$: Poor; $H_{5}$: Terrible.

To represent these subjective terms in a utility context, we present five commonly used fuzzy models from the literature. Each model defines fuzzy numbers, either triangular or trapezoidal, to represent ordinal categories based on different semantic assumptions. Specifically: Utility 1 (see [43,44]), Utility 2 (see [48]), Utility 3 (see [48]), Utility 4 (see [19,49]), and Utility 5 (see [50,51]). These models facilitate the transformation of qualitative assessments into computational structures, a crucial step in constructing the ASI index. Table 6 and Table 7 present the fuzzy number parameters used for each utility model, forming the foundation for converting vague, linguistically rich input into structured data for evaluating airline service quality.

Utility systems U1, U2, and U3 use triangular fuzzy numbers, represented by a triplet (l,m,u), where l is the lower bound, m is the most representative (modal) value, and u is the upper bound. Triangular fuzzy numbers are conceptually simple and computationally efficient, making them well-suited for quick approximations of linguistic assessments. However, they may be limited in representing asymmetric or boundary uncertainty. In contrast, utility systems U4 and U5 use trapezoidal fuzzy numbers, represented by a quadruple (a,b,c,d), where [b,c] defines the core (plateau) of full membership, and [a, b) and (c, d] form the shoulders, indicating gradual transitions. Trapezoidal fuzzy numbers offer greater flexibility, especially when the linguistic judgment includes a range of equally plausible values (i.e., a “flat top”). This is particularly advantageous when modeling less precise or more ambiguous evaluations.

**Figure 8:**
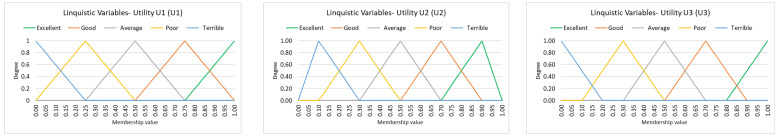
The triangular fuzzy membership functions corresponding to utility models U1, U2, and U3. Source: Author’s elaboration based on Table 6.

**Figure 9:**
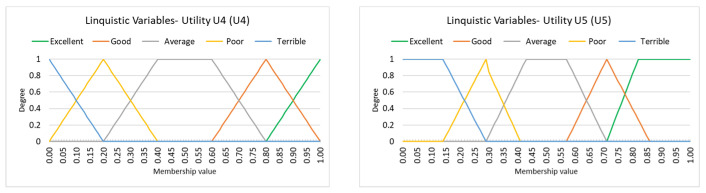
The trapezoidal fuzzy membership functions corresponding to utility models U4, and U5. Source: Author’s elaboration based on Table 7.

The following figures illustrate the membership functions of linguistic variables on a 5-point ordinal scale, modeled using triangular (U1−U3) (Figure 8) and trapezoidal (U4, U5) (Figure 9) fuzzy numbers. These visualizations depict how linguistic variables on a 5-point ordinal scale are mapped using various fuzzy representations, highlighting differences in shape, slope, and support across the modeling approaches.

Below is a comparison of the three triangular fuzzy models (U1, U2, U3) and two trapezoidal models (U4, U5), focusing on their shape, interpretation, and typical use cases.

Utility 1 (U1) is characterized by sharp, optimistic peaks, especially for higher satisfaction grades. For example, “Excellent” starts at 0.75 and peaks at 1. This model assumes high certainty and confidence in ratings, making it suitable for expert reviews or feedback where strong opinions are expected. Utility 2 (U2) provides a more moderate and balanced representation. The transitions between grades are smoother, with broader bases, allowing for greater overlap. This makes U2 ideal for analyzing general user reviews where opinions might be slightly ambiguous or less consistently expressed. This reflects the reality of everyday user reviews, where satisfaction levels are often fuzzy, uncertain, or context-dependent. U2 is well suited for platforms like TripAdvisor, where reviews may vary in tone and certainty. Utility 3 (U3) offers the most distinct boundaries among the triangular models. The transitions are sharper, and high grades start at slightly higher values than U2. While it appears similar to U2 in shape, key differences exist at the boundary (extreme) values. For instance, “Terrible” in U2 starts from (0, 0.1, 0.3), while in U3 it is (0, 0, 0.2); and “Excellent” in U2 spans (0.7, 0.9, 1) or (0.8, 1, 1), whereas in U3 it begins at 0.8 and remains sharper at the right-hand end. These subtle differences at the left and right extremities of the scale make U3 more suitable for applications that require stricter classification or minimal ambiguity at the endpoints. While U2 and U3 may appear visually similar in the middle ranges, the way they treat the edge values (near 0 and 1) has important implications: U2 is more forgiving and human-like, accepting some vagueness at the extremes, while U3 enforces clearer cutoffs, making it better for logic-driven or machine-learning contexts. Utility 4 (U4) uses trapezoidal shapes with wide plateaus, especially for positive ratings. This allows the “Excellent” and “Good” grades to maintain full membership over a broader range. As a result, U4 effectively captures human-like vagueness and is well-suited for summarizing user feedback that may contain soft or imprecise expressions. Utility 5 (U5) is a more refined and conservative model. While still trapezoidal, the plateau regions are narrower, leading to a more precise interpretation. It minimizes the risk of overestimating satisfaction and is appropriate for analytical contexts, such as dashboard visualizations or management reporting, where reliability and caution are valued.

Each fuzzy model offers a different lens for interpreting linguistic input. Triangular models are simpler and better suited for computational efficiency and categorization. In contrast, trapezoidal models better emulate human uncertainty and fuzziness in language, which is valuable for nuanced opinion analysis.

In our study, we employ all five utility functions (U1−U5) and compare the results derived from each as part of a sensitivity analysis. This approach is essential given that we do not have access to detailed respondent-level data, nor do we perform text mining or analyze open-ended textual responses.

Similarity values from the matrices calculated using Formulas (4)–(6) for Utility functions 1–5 are as follows:

(15) $$\tilde{S}U1=\left[\begin{array}{ccccc} 1 & 0.833 & 0.583 & 0.333 & 0.167\\ 0.833 & 1 & 0.750 & 0.500 & 0.333\\ 0.583 & 0.750 & 1 & 0.750 & 0.583\\ 0.333 & 0.500 & 0.750 & 1 & 0.833\\ 0.167 & 0.333 & 0.583 & 0.833 & 1 \end{array}\right],$$

(16) $$\tilde{S}U2=\left[\begin{array}{ccccc} 1 & 0.833 & 0.633 & 0.433 & 0.267\\ 0.833 & 1 & 0.800 & 0.600 & 0.433\\ 0.633 & 0.800 & 1 & 0.800 & 0.633\\ 0.433 & 0.600 & 0.800 & 1 & 0.833\\ 0.267 & 0.433 & 0.633 & 0.833 & 1 \end{array}\right],$$

(17) $$\tilde{S}U3=\left[\begin{array}{ccccc} 1 & 0.767 & 0.567 & 0.367 & 0.133\\ 0.767 & 1 & 0.800 & 0.600 & 0.367\\ 0.567 & 0.800 & 1 & 0.800 & 0.567\\ 0.367 & 0.600 & 0.800 & 1 & 0.767\\ 0.133 & 0.367 & 0.567 & 0.767 & 1 \end{array}\right],$$

(18) $$\tilde{S}U4=\left[\begin{array}{ccccc} 1 & 0.850 & 0.550 & 0.250 & 0.100\\ 0.850 & 1 & 0.700 & 0.400 & 0.250\\ 0.550 & 0.700 & 1 & 0.700 & 0.550\\ 0.250 & 0.400 & 0.700 & 1 & 0.850\\ 0.100 & 0.250 & 0.550 & 0.850 & 1 \end{array}\right],$$

(19) $$\tilde{S}U5=\left[\begin{array}{ccccc} 1 & 0.820 & 0.608 & 0.395 & 0.215\\ 0.820 & 1 & 0.788 & 0.575 & 0.395\\ 0.608 & 0.788 & 1 & 0.788 & 0.608\\ 0.395 & 0.575 & 0.788 & 1 & 0.820\\ 0.215 & 0.395 & 0.608 & 0.820 & 1 \end{array}\right].$$

We adapted the Fuzzy Belief Structure (FBS) approach due to its intuitive nature, computational efficiency, and ability to directly capture the inherent vagueness of ordinal linguistic evaluations. Unlike many group decision-making or text-mining methods, FBS is simple and interpretable, requiring neither consensus-building among experts nor extensive preprocessing of textual data [52,53]. It functions effectively on limited categorical inputs without large training corpora. Importantly, it maintains a faithful representation of linguistic uncertainty, avoiding the information loss that often occurs when qualitative evaluations are vectorized for NLP or ML models. While text-mining and ML approaches excel when rich textual corpora are available, our context focuses on numerical and ordinal social ratings rather than full-text reviews. In this setting, FBS provides a direct, lossless translation of human evaluations into a computational structure suitable for further analysis, such as constructing the ASI index.

## 4. Results and Discussion

In this section, we evaluated customer satisfaction for 25 international Star Alliance airlines through a hybrid analytical framework that integrates both fuzzy logic modeling and multi-criteria evaluation. This approach was designed to capture the inherent uncertainty of subjective feedback often encountered on platforms such as TripAdvisor, especially in contexts where textual reviews are unstructured, unavailable, or not mined in detail.

The first tier of our analysis involves the construction of a series of Airline Satisfaction Indexes (ASI1−ASI5) based on fuzzy representations of linguistic ratings across a 5-point ordinal scale. These indices are generated using five different fuzzy utility functions (U1−U5) —including triangular and trapezoidal fuzzy numbers—to reflect varying assumptions about the shape and strictness of satisfaction levels. This enables us to examine how different interpretations of linguistic terms affect the overall ranking of airlines, allowing for a robust sensitivity analysis across modeling choices.

The second tier incorporates ratings across nine key service dimensions, such as legroom, in-flight entertainment, value for money, check-in and boarding, seat comfort, customer service, cleanliness, food and beverage. These ratings were originally collected in circle-based form and normalized to a numerical 1–5 scale. By combining these aspect-specific scores with fuzzy-based global satisfaction metrics, we gain a more nuanced and actionable understanding of airline performance.

This integrated methodology—grounded in fuzzy logic yet enriched by detailed service evaluations—enables us to achieve the following:

- Assess the robustness of satisfaction rankings under different utility functions
- Identify the most consistently high-performing airlines, and
- Detect latent service gaps across individual criteria.

Together, these insights provide a comprehensive platform for benchmarking airline service quality, particularly in scenarios where traditional quantitative feedback is limited or difficult to interpret. This approach offers a comprehensive understanding of airline performance, particularly valuable in cases where structured textual reviews are unavailable or not subject to text mining techniques.

The Airline Satisfaction Index (ASI) procedure is carried out following the step-by-step procedure detailed in Section 3.3, as summarized below.

Step 1. Data Collection

The set of evaluated objects is 25 Star Alliance airlines, assessed using five linguistic categories H1 (Excellent)–H5 (Terrible). The output data for the subsequent steps of the procedure are presented in Table 2 (Section 3.1), which includes the total number of respondents as well as the number of responses for each grade $Hk\;\left(k\;=\;1,\;2,\;3,\;4,\;5\right)$.

Step 2. Determine the Fuzzy Utility Function and Similarity Matrix

Each satisfaction level is assigned a utility value using Formula (2) for triangular fuzzy numbers or Formula (3) for trapezoidal fuzzy numbers. We adopt the utility functions U1−U5, as presented in Table 6 and Table 7 in Section 3.4. These values are subsequently processed using Formula (4) for U1−U3 and Formula (5) for U4−U5, form the basis for constructing the similarity matrices ($\tilde{S}U1-\tilde{S}U5)$, which are presented in Section 3.4 (Formulas (15)–(19)).

Step 3. Construction of Fuzzy Belief Structures

Respondents’ evaluations are transformed into belief distributions for each airline (see Table 8), by Formula (9). For example, for AEGEAN, the fuzzy belief structure is (according to Formula (8)): FBS($O_{1})\;$= {(Excellent, 0.448), (Good, 0.248), (Average, 0.074), (Poor, 0.043), (Terrible, 0.187)}. This is represented as a belief vector: S1 = [0.448, 0.248, 0.074, 0.043, 0.187], as defined in Formula (9).

Step 4. Identify the Ideal and the Negative-Ideal Belief Solutions

The Positive Ideal Belief Solution (PIBS) represents full satisfaction: $A^{+}=\left[1,\;0,\;0,\;0,\;0\right]$, while the Negative Ideal Belief Solution (NIBS) represents full dissatisfaction: $A^{-}=\left[0,\;0,\;0,\;0,\;1\right]$. Full satisfaction occurs when all respondents (100%) select the rating category corresponding to “Excellent,” whereas full dissatisfaction is observed when all (100%) respondents choose the “Terrible” category.

Step 5. Compute Separation Measures

Distances from each airline’s belief vector to PIBS and NIBS are calculated using belief-based distance measures (Formulas (12) and (13)), taking into account $\tilde{S}U1-\tilde{S}U5$ (Formulas (15)–(19)). For example, for AEGEAN under $\tilde{S}U1$, the distance to PIBS is 0.305 and to NIBS is 0.642, while under $\tilde{S}U5$ the distance to PIBS is 0.303, and to NIBS is 0.619.

Step 6. Calculate the Relative Closeness

Relative closeness to the ideal solution is calculated using the standard TOPSIS closeness coefficient (Formula (14)). For AEGEAN, the values are 0.678 for ASI1 and 0.671 for ASI5. The results for all airlines are presented in Table 9. Higher closeness values indicate greater satisfaction.

Step 7. Rank the Objects

Finally, the airlines are ranked based on their relative closeness scores. Table 9 presents the rankings corresponding to ASI1−ASI5. The last two columns show the general TripAdvisor rating based on the average score (TA) (see Table 2) and the corresponding position in the overall ranking.

**Figure 10:**
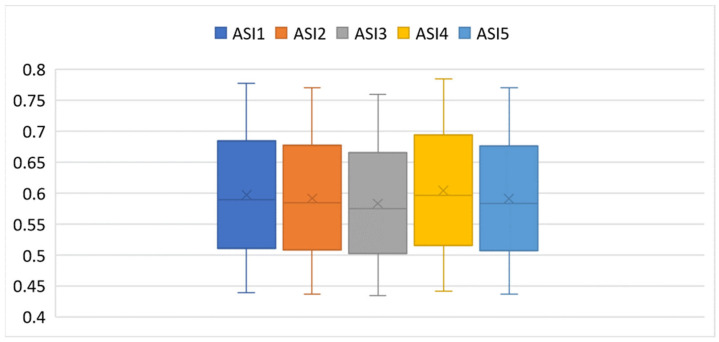
Box plots of ASI1–ASI5 scores across Star Alliance airlines, based on the data from Table 9.

To better illustrate the variability and distribution of the ASI scores shown in Table 9, Figure 10 presents boxplots for ASI1-ASI5.

The results presented in Table 9 and visualized in Figure 10 show that satisfaction scores across the 25 Star Alliance airlines are generally clustered, with only minor differences across the five Airline Satisfaction Index variants (ASI1–ASI5). Average values range from 0.583 (ASI3) to 0.604 (ASI4), indicating that despite varying fuzzy modeling approaches, the overall satisfaction structure remains stable. Among the five models, ASI4 yields the highest mean (0.604) and the greatest variability (standard deviation = 0.102, range = 0.344), suggesting it enables more expressive differentiation, from high performers like Singapore Airlines (0.785) to low scorers such as Air India (0.441). This wider spread reflects greater sensitivity to both positive and negative sentiment. In contrast, ASI3 has the lowest mean (0.583) and the most compact distribution (standard deviation = 0.096, range = 0.326). While slightly more conservative, it offers stronger internal coherence and reduced influence from outliers.

The interquartile ranges (IQRs) for all ASIs are relatively narrow (typically 0.06–0.07), confirming that most airlines are concentrated near the median, with only a few outliers affecting the extremes. This reinforces the view of a compact satisfaction structure across the alliance. ASI1,ASI2, and ASI5 are nearly identical in central tendency and dispersion, often differing by less than 0.01 per airline. Their strong alignment indicates high internal consistency.

In summary, ASI3 emerges as the most stable and internally consistent model, ideal for highlighting consensus and central trends. In contrast, ASI4 offers broader differentiation, making it more suitable for detecting marginal or nuanced differences in satisfaction level.

This conclusion is supported by Pearson and Spearman correlation coefficients (Table 10): Pearson correlations range from 0.9993 to 1.0000, indicating nearly perfect linear agreement, while Spearman rank correlations (0.9984 to 1.0000) confirm the stability of airline rankings across models.

Singapore Airlines consistently holds the top position in the rankings, regardless of the ASI variant. Air New Zealand and ANA (All Nippon Airways) follow in second and third place, alternating positions depending on the ASI model: ANA ranks second in ASI1 and ASI4, while Air New Zealand occupies that position in the remaining configurations. All three airlines maintain ASI scores above 0.70 across all models, reaffirming their status as industry leaders in customer satisfaction. Singapore Airlines and Air New Zealand stand out across all rating categories, each earning top scores of 4.5 in Cleanliness, Check-in and boarding, and Customer service. ANA particularly excels in the cleanliness category, further reinforcing its strong performance and high customer satisfaction.

Just behind the leaders, EVA Air and Asiana Airlines consistently secure fourth and fifth positions across all five indexes. EVA Air’s ASI scores range from 0.740 (ASI3) to 0.772 (ASI4), while Asiana Airlines’ scores vary from 0.679 (ASI3) to 0.712 (ASI4). Both carriers demonstrate strong consistency in category-based evaluation (Table 5). EVA Air earns excellent ratings of 4.5 in Cleanliness and Check-in and boarding, and solid scores of 4.0 in all other categories. Asiana Airlines also maintains high scores of 4.0 across all dimensions except In-flight entertainment, where it receives a slightly lower rating of 3.5. These results translate into a strong and cohesive passenger experience for both airlines.

Thai Airways rounds out the top six, with ASI scores ranging from 0.669 (ASI3) to 0.701 (ASI4). The airline performs well across all categories (Table 5), although it scores slightly lower (3.5) in In-flight entertainment.

In the mid-tier segment, carriers such as Lufthansa, Austrian Airlines, and Shenzhen Airlines deliver solid but less distinctive performance. Lufthansa’s ASI scores range from 0.575 (ASI3) to 0.596 (ASI4), Austrian Airlines from 0.567 (ASI3) to 0.587 (ASI4), and Shenzhen Airlines from 0.553 (ASI3) to 0.576 (ASI4). These airlines consistently occupy the 13th to 15th positions across all ASI variants. While they perform well in key satisfaction categories such as Cleanliness, they lack strong differentiators in In-flight entertainment, which likely contributes to their middle-ranking positions.

At the bottom of the ranking, Air India, TAP Air Portugal, LOT Polish Airlines, and Air Canada consistently occupy the last four positions. Air India ranks 25th across all ASI variants, with scores ranging from 0.434 (ASI3) to 0.441 (ASI4), indicating a persistently low level of passenger satisfaction. The ordinal ratings reveal significant weaknesses across all categories except Legroom, which received a relatively better score of 3.5. TAP Air Portugal scores between 0.460 (ASI3) and 0.472 (ASI4), while LOT ranges from 0.464 (ASI3) to 0.474 (ASI4), placing 24th and 23rd, respectively. Both airlines perform reasonably well in terms of Cleanliness and Check-in and boarding, but fall short in key areas such as Customer service, Seat comfort, and In-flight entertainment—critical aspects for long-haul travel. Air Canada, with ASI scores from 0.481 (ASI3) to 0.492 (ASI4), ranks 22nd and shows a similarly underwhelming profile to the other carriers at the lower end of the list.

Although 19 airlines maintained stable positions across all five ASI configurations, a few mid-ranking carriers experienced minor shifts, typically by just one position. These slight fluctuations, seen in the cases of South African Airways, Turkish Airlines, Ethiopian Airlines, and United Airlines, can be linked to how different aspects of satisfaction ratings, especially those in the mid-range, are interpreted by different fuzzy models. South African Airways, for instance, received mostly average scores (3–3.5) across all categories, which led to marginal downgrades in models that handle moderate evaluations more cautiously. Turkish Airlines, with a mix of 3.5 s and some 4 s in service-related areas, improved slightly in more optimistic models but lost ground where differences in criteria ratings were smoothed out. Ethiopian Airlines, with ratings not exceeding 3.5 across all criteria, dropped one position in a model that differentiated more sharply within the mid-range. United Airlines, despite having a similarly average profile, performed slightly better only in areas like Check-in and boarding, which may have contributed to a minor gain under those same conditions.

These cases illustrate the impact of fuzzy utility interpretation on data and underscore the importance of sensitivity analysis in ensuring that rankings remain fair and robust, illustrate the impact of fuzzy utility interpretation on data and underscore the importance of sensitivity analysis in ensuring that rankings remain fair and robust, even when based on nuanced linguistic inputs.

In the comparison of positions between the ASI rankings and the ranking based on average ratings from TripAdvisor (TA in Table 9), as many as 17 airlines held the same place. The remaining airlines showed slight differences in positions, which did not exceed two places. The largest deviations were observed in the rankings of Air New Zealand, which ranks 2nd in ASI2,ASI3,ASI5, but 4th in TripAdvisor, and South African Airways, ranked 12th in ASI2,ASI3,ASI5 and 10th in TripAdvisor. EVA Air was rated one position higher in the TripAdvisor ranking compared to ASI. Avianca and Ethiopian Airlines also showed a one-place difference, favoring either ASI. This high consistency of the rankings is also reflected in the very high Spearman correlation, which for all five ASI variants compared to the TripAdvisor ranking ranges from 0.995 to 0.998. These results confirm that the ASI method very accurately reflects user opinions available on the TripAdvisor platform, and the minor differences in positions mainly stem from natural variations in the way ratings are aggregated and data modeled.

Although the results produced by the proposed Airline Satisfaction Index closely align with traditional average-based ratings (TA measure), the ASI method offers several important advantages that enhance both its practical application and methodological soundness. One of the core strengths of ASI is its ability to account for the uncertainty and vagueness present in user-generated reviews. Traditional arithmetic means rely on the assumption that ordinal categories, such as “Good” and “Excellent,” are evenly spaced and precisely defined. ASI, in contrast, models user feedback through fuzzy belief distributions, capturing the imprecision, subjectivity, and nuance that characterize real-world linguistic evaluations. This capacity becomes particularly relevant in the context of modern review platforms, which operate as decentralized and socially influenced opinion networks. User feedback in these environments is dynamic, diverse, and often inconsistent, and the fuzzy logic foundation of ASI aligns naturally with the complex and fluid nature of this data. In addition to its conceptual alignment with the structure of online reviews, ASI demonstrates robustness across varying modeling assumptions. By applying several distinct fuzzy utility functions, the method consistently produces stable airline rankings, even when the underlying assumptions about utility interpretation differ. This stability indicates that ASI is not overly dependent on subjective parameter choices, which strengthens confidence in its objectivity and reliability. Another valuable feature of the ASI framework is its heightened sensitivity to performance variation, particularly in the case of airlines with mid-range ratings. While ASI rankings are generally in line with those generated through arithmetic averages, certain configurations of the index are more responsive to subtle differences in service quality. This allows ASI to uncover distinctions that might otherwise be obscured in traditional approaches, leading to more detailed and informative evaluations. The validity of the method is confirmed through very high Pearson and Spearman correlation coefficients between ASI and TripAdvisor rankings. Crucially, this high degree of consistency is achieved without sacrificing the added value of representing fuzziness, which provides an important methodological enhancement over conventional averaging techniques.

Finally, while Table 2 and Table 9 suggest a clear relationship between entropy levels and customer satisfaction, this association should not be interpreted as deterministic. Airlines with lower entropy values often achieve higher ASI ratings, reflecting more consistent and generally positive passenger feedback. Conversely, higher entropy is typically associated with lower satisfaction and more divergent opinions. However, this correlation arises from the specific distributional patterns of customer ratings and should not be regarded as a universal rule.

Entropy is a measure of inconsistency—not sentiment. It quantifies the uncertainty or dispersion within the feedback but does not reveal whether the prevailing opinions are mostly positive or negative. For instance, two airlines might share the same entropy value: one could be polarized, with ratings clustered at the extremes such as “Excellent” and “Terrible”, while another might reflect consistent mediocrity, with ratings evenly spread across all categories. In both cases, entropy would be high, yet the nature of passenger perception would differ significantly.

As such, relying on entropy alone to rank service quality is insufficient. A valid interpretation of customer satisfaction requires a combined analysis of entropy and the underlying structure of the rating distribution. Entropy offers insight into the coherence and reliability of passenger experiences, but not into their overall sentiment or direction. To accurately assess service quality, one must consider the full distribution of ratings, taking into account the presence of patterns such as bimodality, skewness, or uniformity, and interpret these alongside average satisfaction scores within the broader context of customer feedback. Only through such a comprehensive, distribution-sensitive approach can we uncover the true structure of customer perception, enabling more accurate benchmarking and more effective strategies for service improvement.

Based on the findings, the analysis successfully addressed all five research questions. The synthesized response could read as follows:

- The results confirmed that Star Alliance airlines’ satisfaction rankings remain highly robust across different fuzzy utility models. Despite variations in the shape of the utility functions, the ranking structure remained largely unchanged. Nineteen airlines held identical positions across all models, and any observed differences were minimal. Very high Pearson and Spearman correlation coefficients further support the consistency and reliability of the fuzzy modeling approach.
- The analysis also made it possible to identify Star Alliance airlines that consistently deliver high levels of satisfaction. Singapore Airlines, Air New Zealand, and ANA ranked at the top across all configurations, with EVA Air, Asiana Airlines, and Thai Airways also demonstrating strong and stable performance. Their consistently high scores across all service dimensions reflect reliable service quality regardless of the modeling technique applied.
- In comparing the fuzzy-based indexes with traditional average ratings, the results showed a strong level of agreement. Seventeen airlines were ranked in the same position in both the ASI and TripAdvisor-based rankings, while deviations among the remaining carriers did not exceed two places. The very high Spearman correlations between the methods confirm that the ASI approach aligns closely with conventional evaluations, supporting its validity.
- The TripAdvisor airline data enabled the identification of key service dimensions that differentiate airline performance. High-ranking carriers excelled in areas such as Cleanliness, Check-in and boarding, and Customer service. In contrast, lower-ranked Star Alliance airlines, including Air India, TAP Air Portugal, and LOT Polish Airlines, showed consistent weaknesses across several categories, particularly in Customer service and In-flight entertainment. The models’ sensitivity to category inputs also revealed subtle distinctions among mid-tier airlines, illustrating the added value of this approach in capturing nuanced passenger experiences.
- Finally, entropy classes show a clear relationship with average and ASI customer satisfaction rankings. Airlines in Entropy Class I consistently rank highest on TripAdvisor and ASI ratings, indicating stable service excellence. In contrast, Entropy Class IV airlines are typically at the bottom of satisfaction rankings, with highly inconsistent feedback. Airlines in Entropy Classes II and III show moderate entropy and occupy middle-tier positions.

Table 11 presents a comparative overview of different approaches for evaluating airline service quality, highlighting the results obtained using the ASI (Adapted TOPSIS with Fuzzy Belief Structure), the traditional arithmetic mean (TA), and entropy-based analysis.

## 5. Conclusions

This study introduces a hybrid framework combining Fuzzy Belief Structures with the TOPSIS method to assess airline service quality using ordinal, user-generated reviews from TripAdvisor. By transforming linguistic evaluations into fuzzy belief distributions, the model allows for robust and interpretable airline benchmarking under uncertainty. The five ASI variants, based on different fuzzy utility functions, consistently identified top-performing airlines such as Singapore Airlines, ANA, EVA Air, and Air New Zealand, while also highlighting key service dimensions like entertainment and seat comfort as drivers of differentiation. Despite small variations in rankings, the high correlations among ASIs confirm the stability and reliability of the approach.

In sum, the ASI framework offers a scalable, transparent, and theoretically grounded approach to modeling feedback in complex social systems, providing valuable insights for researchers, airline managers, and digital platform designers alike. By combining methodological rigor with a realistic representation of subjective user feedback, the ASI approach serves as a robust and nuanced enhancement over traditional average-based rating systems (TA), particularly in handling subjective, imprecise, and complex data.

Furthermore, entropy analysis reveals a strong link between the diversity of customer opinions and both ASI and TripAdvisor rankings, confirming that service consistency is a key driver of airline reputation.

The adapted TOPSIS with Fuzzy Belief Structure in our ASI framework offers several strengths. Using belief-based distance measures allows nuanced differentiation between airlines even when ratings are close, while integrating uncertainty modeling directly into the distance computation. This approach produces stable rankings across different fuzzy utility assumptions. However, it is slightly more computationally intensive than simple averaging or entropy-only analysis and is sensitive to the choice of fuzzy membership functions, although our results show high robustness with proper initial calibration. Compared with the arithmetic mean, as used in TripAdvisor’s TA measure, our method captures subtle differences in mid-tier performance that simple averages tend to mask. Compared with entropy-only analysis, ASI incorporates both performance level and rating stability, whereas entropy alone measures only variability. Other MCDM methods, such as fuzzy VIKOR and fuzzy AHP, could also be applied, but they typically require more complex preference elicitation, which may be less transparent to practitioners than TOPSIS’s intuitive relative closeness interpretation. It should be noted that the MCDM approach can also be applied in a non-standard, single-criterion context, i.e., the overall satisfaction rating provided by users.

While the framework proves effective, it is limited by its focus exclusively on Star Alliance airlines and a single data source (TripAdvisor), which may limit generalizability as well as the exclusion of textual data. Future research could address these gaps by applying the model to broader datasets, incorporating sentiment analysis, and developing more adaptive or data-driven utility functions. Moreover, further exploration into the networked structure of opinion formation, such as how reviews propagate and influence future evaluations, would deepen the understanding of satisfaction dynamics in digital environments. Comparative studies with alternative decision-making methods and applications in other service sectors, such as hospitality or public transport, could further validate and expand the approach. In addition, future work could explore alternative entropy-based measures to capture different dimensions of rating variability and information uncertainty.

## Figures and Tables

**Table 1:** Overview of Selected Studies on Airline Service Quality.

Authors	Method Used	Focus Area/ Variables Studied	Key Findings
[7]	Weighted SERVQUAL	Perception vs. expectation (Turkey)	Responsiveness most important; varies by passenger profile
[8]	SERVQUAL + Kano	Cross-cultural perceptions (USA and Turkey)	Perceptions below expectations; differences in priorities
[9]	Fuzzy AHP + TOPSIS	Fuzzy logic for intangible criteria	Courtesy, safety, comfort most valued
[10]	Best-Worst Method + VIKOR	Prioritization of service quality attributes (India)	Tangibility, reliability, safety most valued
[11]	MUSA (MCDM)	Multicriteria passenger satisfaction	Identified dimensions for targeted service improvement
[12]	Synthetic Measure for Ordinal Data (SMOD)	Star Alliance airline ratings on TripAdvisor	Singapore Airlines rated best; SMOD discriminates well
[13]	Latent Semantic Analysis	Drivers of satisfaction/dissatisfaction	Class- and carrier-type-dependent preferences
[14]	Deep learning (BERT), sentiment analysis	TripAdvisor reviews, COVID-19 impact	Deep learning effective for aspect-based analysis
[15]	Correspondence analysis	Relative positioning of US airlines	Positioning varies by objective DOT criteria
[16]	Statistical + Cultural Analysis	Domestic bias in ratings	Higher scores for domestic airlines; influenced by culture
[27]	Structural Equation Modeling (SEM), SERVPERF	Linking service quality, satisfaction, behavioral intentions	Schedule most influential; satisfaction leads to loyalty
[28]	Text mining, Latent Dirichlet Allocation (LDA), regression analysis	Customer satisfaction dimensions from 55,000+ online reviews; variables: passenger type, cabin class, satisfaction dimensions	Identified 27 satisfaction dimensions predicting airline recommendation with 79.95% accuracy; cabin staff, onboard service, and value for money most influenced recommendations; customer service focus should vary by cabin class.
[29]	Machine learning classifiers	Predicting customer return visits using feedback comments and satisfaction ratings	Achieved 83.42% accuracy in predicting return visits; longer feedback improves prediction accuracy.
[30]	Structural Topic Models (STM), combining textual and numerical data	Airline customer satisfaction; service quality factors from online reviews	Combining textual and numerical data improves satisfaction prediction; reveals key service drivers and explains low-cost carriers’ success.
[31]	Latent Dirichlet allocation (LDA) sentiment analysis	Online reviews of 27 Asian airlines; key service aspects like seat, service, meal, delays	Seat, service, and meal were key topics; delays caused dissatisfaction, while staff service increased satisfaction.
[32]	Semantic network analysis (CONCOR), linear regression	Customer satisfaction and recommendation; evaluation factors: seat comfort, staff, F&B, entertainment, ground service, value for money	All factors except entertainment significantly influenced satisfaction and recommendation; online reviews offer valuable strategic insights for the airline industry.

Source: Author’s elaboration.

**Table 2:** Overall ratings of Star Alliance airlines.

No.	Airline	Numbers of Reviews	Linguistic Evaluation with a 5-Point Ordinal Scale					Average Score	Shannon Entropy
			Excellent	Good	Average	Poor	Terrible		
1	AEGEAN	14,295	6403	3540	1063	614	2675	3.726	1.944
2	Air Canada	32,133	6273	7554	5189	3618	9499	2.922	2.250
3	Air China	6533	988	1757	1460	843	1485	2.988	2.272
4	Air India	10,941	1751	2367	1692	1191	3940	2.707	2.196
5	Air New Zealand	14,404	7921	3349	1288	698	1148	4.124	1.778
6	ANA (All Nippon Airways)	10,771	5448	3280	1046	419	578	4.170	1.755
7	Asiana Airlines	3797	1492	1242	531	194	338	3.884	1.984
8	Austrian Airlines	9957	3018	2612	1201	764	2362	3.317	2.173
9	Avianca	22,109	7120	6326	2367	1394	4902	3.424	2.121
10	Brussels Airlines	8585	1718	2744	1359	752	2012	3.164	2.210
11	Copa Airlines	13,912	4317	4259	2196	1033	2107	3.550	2.158
12	Croatia Airlines	1246	281	362	211	121	271	3.209	2.242
13	EGYPTAIR	5244	1015	1292	908	482	1547	2.952	2.231
14	Ethiopian Airlines	7069	1392	1966	1208	662	1841	3.057	2.236
15	EVA Air	7486	3815	2223	602	291	555	4.129	1.769
16	LOT Polish Airlines	6533	1261	1482	955	660	2175	2.846	2.211
17	Lufthansa	43,887	13,748	11,661	5090	3204	10,184	3.355	2.158
18	Shenzhen Airlines	346	64	114	79	35	54	3.286	2.217
19	Singapore Airlines	27,262	15,497	6136	2550	1180	1899	4.179	1.731
20	South African Airways	4998	1197	1620	996	500	685	3.429	2.210
21	Swiss International Air Lines [SWISS]	16,586	5695	4309	1921	1283	3378	3.462	2.148
22	TAP Air Portugal	25,519	4809	6105	3366	2431	8808	2.831	2.186
23	Thai Airways	16,772	6284	5558	2394	1021	1515	3.839	2.019
24	Turkish Airlines	38,433	14,215	8645	3331	2313	9929	3.388	2.069
25	United Airlines	56,701	11,366	14,211	10,823	7145	13,156	3.061	2.287

Source: Author’s elaboration based on Tripadvisor data [35] (accessed on 12 July 2025).

**Table 3:** Descriptive statistics for the distribution of linguistic ratings for Star Alliance airlines.

Descriptive Statistics/Category	Excellent	Good	Average	Poor	Terrible
Min	0.151	0.216	0.074	0.039	0.054
Max	0.568	0.331	0.228	0.129	0.360
Mean	0.306	0.272	0.141	0.080	0.202
Standard deviation	0.127	0.037	0.042	0.027	0.089
Variability	0.416	0.135	0.301	0.334	0.440

Source: Author’s elaboration based on Tripadvisor data [35] (accessed on 12 July 2025).

**Table 4:** Entropy-Based Classification of Airline Ratings.

Entropy Class	Core Characteristics	Airlines	Strategic Focus
Class I: Very Low Entropy [1.731; 2.019)	High satisfaction and consistency. Ratings are strongly peaked around “Excellent” and “Good”, with minimal presence in negative categories. Occasionally bimodal but low in mid-tier reviews.	AEGEAN, Air New Zealand, ANA, Asiana Airlines, EVA Air, Singapore Airlines, Thai Airways	Maintain service excellence, promote loyalty, and sustain consistency.
Class II: Low Entropy [2.019; 2.173)	Generally positive feedback with some inconsistency. A few outlier negative reviews slightly raise entropy, suggesting variability in perception rather than actual dissatisfaction.	Austrian Airlines, Avianca, Copa Airlines, Lufthansa, SWISS, Turkish Airlines	Monitor feedback trends; ensure quality control across service segments.
Class III: Moderate Entropy [2.173; 2.217)	Mixed perception across the airlines. Balanced distribution of ratings, often with a notable share in both positive and negative categories. Indicates divided customer experiences or uneven service quality.	Air India, Brussels Airlines, LOT Polish Airlines, Shenzhen Airlines, South African Airways, TAP Air Portugal	Audit service delivery; train staff and clarify brand expectations.
Class IV: High Entropy [2.217; 2.287]	High variability in feedback with considerable weight in negative categories. Ratings spread across all classes, including strong presence of “Bad” and “Terrible” reviews. Often reflects inconsistent service or polarized experiences.	Air Canada, Air China, Croatia Airlines, EGYPTAIR, Ethiopian Airlines, United Airlines	Undertake deep operational reviews; stabilize customer experience.

Source: Author’s elaboration based on Tripadvisor data [35] (accessed on 12 July 2025).

**Table 5:** TripAdvisor ratings of Star Alliance airlines by criterion and overall score.

No	Airline	C1	C2	C3	C4	C5	C6	C7	C8	C9	General Rating
1	AEGEAN	3.5	3.5	2.5	4	4	4	4	4	4	3.5
2	Air Canada	3.5	3	3	3	3	3	3.5	3.5	3	3
3	Air China	3	3	2.5	3	3	3.5	3.5	3	3	3
4	Air India	3.5	3	2.5	3	3	3	3	3	3	2.5
5	Air New Zealand	4	4	4	4	4.5	4	4.5	4.5	4	4
6	ANA (All Nippon Airways)	4	4	4	4	4	4	4.5	4	4	4
7	Asiana Airlines	4	4	3.5	4	4	4	4	4	4	4
8	Austrian Airlines	3.5	3.5	3	3.5	3.5	3.5	4	3.5	3.5	3.5
9	Avianca	3.5	3.5	3.5	3.5	3.5	3.5	4	3.5	3.5	3.5
10	Brussels Airlines	3.5	3.5	2.5	3	3.5	3.5	3.5	3.5	3	3
11	Copa Airlines	3.5	3.5	3.5	3.5	4	3.5	4	4	3.5	3.5
12	Croatia Airlines	3.5	3.5	2	3.5	3.5	3.5	4	3.5	3	3
13	EGYPTAIR	3.5	3	2.5	3	3	3	3	4.5	3	3
14	Ethiopian Airlines	3.5	3.5	3	3	3.5	3.5	3.5	3	3	3
15	EVA Air	4	4	4	4	4	4	4.5	4.5	4	4
16	LOT Polish Airlines	3.5	3.5	2.5	3	3	3	3.5	3.5	3	3
17	Lufthansa	3.5	3.5	3.5	3.5	3.5	3.5	4	3.5	3.5	3.5
18	Shenzhen Airlines	3.5	3.5	2.5	3	3.5	3.5	3.5	3.5	3	3.5
19	Singapore Airlines	4	4	4	4	4.5	4	4.5	4.5	4	4
20	South African Airways	3.5	3.5	3	3	3.5	3.5	4	3.5	3.5	3.5
21	Swiss International Air Lines [SWISS]	3.5	3.5	3	3.5	3.5	3.5	4	4	3.5	3.5
22	TAP Air Portugal	3	3	2.5	3	3	3	3.5	3.5	3	3
23	Thai Airways	4	4	3.5	4	4	4	4	4	4	4
24	Turkish Airlines	3.5	3.5	4	4	3.5	3.5	4	3.5	4	3.5
25	United Airlines	3	3	3	3	3.5	3	3.5	3.5	3	3
	min	3	3	2	3	3	3	3	3	3	
	max	4	4	4	4	4.5	4	4.5	4.5	4	

Source: Based on Tripadvisor data [35] (accessed on 12 July 2025).

**Table 6:** Fuzzy triangular representations of linguistic variables on a 5-point ordinal scale based on selected literature approaches.

Grade	Utility 1 (U1)	Utility 2 (U2)	Utility 3 (U3)
$H_{1}$: Excellent	$U1(H_{1})=(0.75,\;1,\;1)$	$U2(H_{1})=(0.7,\;0.9,\;1)$	$U3(H_{1})=(0.8,\;1,\;1)$
$H_{2}$: Good	$U1(H_{2})=(0.5,\;0.75,\;1)$	${U2(H}_{2)}=(0.5,\;0.7,\;0.9)$	${U3(H}_{2)}=(0.5,\;0.7,\;0.9)$
$H_{3}$: Average	${U1(H}_{3})=(0.25,\;0.5,\;0.75)$	${U2(H}_{3})=(0.3,\;0.5,\;0.7)$	${U3(H}_{3})=(0.3,\;0.5,\;0.7)$
$H_{4}$: Poor	${U1(H}_{4})=(0,\;0.25,\;0.5)$	${U2(H}_{4})=(0.1,\;0.3,\;0.5)$	${U3(H}_{4})=(0.1,\;0.3,\;0.5)$
$H_{5}$: Terrible	$U1(H_{5})=(0,\;0,\;0.25)$	$U2(H_{5})=(0,\;0.1,\;0.3)$	$U3(H_{5})=(0,\;0,\;0.2)$

**Table 7:** Fuzzy trapezoidal representations of linguistic variables on a 5-point ordinal scale based on selected literature approaches.

Grade	Utility 4 (U4)	Utility 5 (U5)
$H_{1}$: Excellent	$U4(H_{1})=(0.8,\;1,\;1,\;1)$	$U5(H_{1})=(0.71,\;0.86,\;1,\;1)$
$H_{2}$: Good	$U4(H_{2})=(0.6,\;0.8,\;0.8,\;1)$	$U5(H_{2})=(0.57,\;0.71,\;0.71,\;0.86)$
$H_{3}$: Average	$U4(H_{3})=(0.2,\;0.4,\;0.6,\;0.8)$	${U5(H}_{3})=(0.29,\;0.43,\;0.57,0.71)$
$H_{4}$: Poor	$U4(H_{4})=(0,\;0.2,\;0.2,\;0.4)$	${U5(H}_{4})=(0.14,\;0.29,\;0.29,\;0.43)$
$H_{5}$: Terrible	$U4(H_{5})=(0,\;0,\;0,\;0.2)$	$U5(H_{5})=(0,\;0,\;0.14,\;0.29)$

**Table 8:** The belief vectors describing the distribution of perceived service quality of Star Alliance airlines.

i	$O_{i}$	$\beta_{i1}$	$\beta_{i2}$	$\beta_{i3}$	$\beta_{i4}$	$\beta_{i5}$
1	AEGEAN	0.448	0.248	0.074	0.043	0.187
2	Air Canada	0.195	0.235	0.161	0.113	0.296
3	Air China	0.151	0.269	0.224	0.129	0.227
4	Air India	0.160	0.216	0.155	0.109	0.360
5	Air New Zealand	0.550	0.233	0.089	0.048	0.080
6	ANA (All Nippon Airways)	0.506	0.304	0.097	0.039	0.054
7	Asiana Airlines	0.393	0.327	0.140	0.051	0.089
8	Austrian Airlines	0.303	0.262	0.121	0.077	0.237
9	Avianca	0.322	0.286	0.107	0.063	0.222
10	Brussels Airlines	0.200	0.320	0.158	0.088	0.234
11	Copa Airlines	0.310	0.306	0.158	0.074	0.152
12	Croatia Airlines	0.226	0.291	0.169	0.097	0.217
13	EGYPTAIR	0.194	0.246	0.173	0.092	0.295
14	Ethiopian Airlines	0.197	0.278	0.171	0.094	0.260
15	EVA Air	0.510	0.297	0.080	0.039	0.074
16	LOT Polish Airlines	0.193	0.227	0.146	0.101	0.333
17	Lufthansa	0.313	0.266	0.116	0.073	0.232
18	Shenzhen Airlines	0.185	0.330	0.228	0.101	0.156
19	Singapore Airlines	0.568	0.225	0.094	0.043	0.070
20	South African Airways	0.240	0.324	0.199	0.100	0.137
21	Swiss International Air Lines [SWISS]	0.343	0.260	0.116	0.077	0.204
22	TAP Air Portugal	0.189	0.239	0.132	0.095	0.345
23	Thai Airways	0.375	0.331	0.143	0.061	0.090
24	Turkish Airlines	0.370	0.225	0.087	0.060	0.258
25	United Airlines	0.200	0.251	0.191	0.126	0.232

Source: Author’s elaboration based on Tripadvisor data [35] (accessed on 12 July 2025).

**Table 9:** The Star Alliance airlines’ rating and ranking for ASI1−ASI5, and the general rating from the TripAdvisor website based on average score (TA).

No	Airline	ASI1	Rank ASI1	ASI2	Rank ASI2	ASI3	Rank ASI3	ASI4	Rank ASI4	ASI5	Rank ASI5	TA	Rank TA
1	AEGEAN	0.678	7	0.672	7	0.662	7	0.686	7	0.671	7	3.726	7
2	Air Canada	0.488	22	0.485	22	0.481	22	0.492	22	0.485	22	2.922	22
**3**	Air China	0.503	20	0.500	20	0.495	20	0.507	20	0.500	20	2.988	20
4	Air India	0.439	25	0.437	25	0.434	25	0.441	25	0.437	25	2.707	25
5	Air New Zealand	0.764	3	0.758	2	0.748	2	0.773	3	0.757	2	4.124	4
6	ANA (All Nippon Airways)	0.766	2	0.757	3	0.744	3	0.778	2	0.757	3	4.170	2
7	Asiana Airlines	0.701	5	0.692	5	0.679	5	0.712	5	0.691	5	3.884	5
8	Austrian Airlines	0.580	14	0.575	14	0.567	14	0.587	14	0.575	14	3.317	14
9	Avianca	0.606	10	0.599	10	0.590	10	0.614	10	0.599	10	3.424	11
10	Brussels Airlines	0.545	17	0.539	17	0.530	17	0.553	17	0.539	17	3.164	17
11	Copa Airlines	0.629	8	0.622	8	0.612	8	0.638	8	0.621	8	3.550	8
12	Croatia Airlines	0.554	16	0.549	16	0.541	16	0.560	16	0.548	16	3.209	16
13	EGYPTAIR	0.496	21	0.492	21	0.487	21	0.501	21	0.492	21	2.952	21
14	Ethiopian Airlines	0.521	18	0.516	18	0.509	19	0.526	18	0.516	18	3.057	19
15	EVA Air	0.761	4	0.753	4	0.740	4	0.772	4	0.752	4	4.129	3
16	LOT Polish Airlines	0.471	23	0.468	23	0.464	23	0.474	23	0.468	23	2.846	23
17	Lufthansa	0.589	13	0.584	13	0.575	13	0.596	13	0.583	13	3.355	13
18	Shenzhen Airlines	0.568	15	0.562	15	0.553	15	0.576	15	0.561	15	3.286	15
19	Singapore Airlines	0.777	1	0.770	1	0.760	1	0.785	1	0.770	1	4.179	1
20	South African Airways	0.599	11	0.592	12	0.582	12	0.607	11	0.592	12	3.429	10
21	Swiss International Air Lines [SWISS]	0.613	9	0.607	9	0.599	9	0.620	9	0.607	9	3.462	9
22	TAP Air Portugal	0.468	24	0.465	24	0.460	24	0.472	24	0.465	24	2.831	24
23	Thai Airways	0.690	6	0.682	6	0.669	6	0.701	6	0.681	6	3.839	6
24	Turkish Airlines	0.598	12	0.594	11	0.586	11	0.604	12	0.593	11	3.388	12
25	United Airlines	0.519	19	0.516	19	0.511	18	0.523	19	0.515	19	3.061	18
	Min	0.439		0.437		0.434		0.441		0.437		2.707	
	Max	0.777		0.770		0.760		0.785		0.770		4.179	
	Mean	0.597		0.591		0.583		0.604		0.591		3.400	
	Standard deviation	0.100		0.098		0.096		0.102		0.098		0.441	
	Variability	0.167		0.166		0.164		0.169		0.166		0.130	

Source: Author’s elaboration based on Tripadvisor data [35] (accessed on 12 July 2025).

**Table 10:** Pearson and Spearman correlation coefficients for ASI variants.

Pearson	ASI1	ASI2	ASI3	ASI4	ASI5	Spearman	ASI1	ASI2	ASI3	ASI4	ASI5
ASI1	1.0000					ASI1	1.0000				
ASI2	0.9999	1.0000				ASI2	0.9984	1.0000			
ASI3	0.9997	0.9999	1.0000			ASI3	0.9977	0.9992	1.0000		
ASI4	0.9999	0.9997	0.9993	1.0000		ASI4	1.0000	0.9984	0.9977	1.0000	
ASI5	0.9999	1.0000	0.9999	0.9997	1.0000	ASI5	0.9985	1.0000	0.9992	0.9985	1.0000

Source: Author’s elaboration.

**Table 11:** Comparison of Methods for Evaluating Airline Service Quality: ASI, TA, and Entropy Analysis.

Method	Key Strengths	Key Limitations
ASI (Adapted TOPSIS with Fuzzy Belief Structure)	Preserves linguistic meaning via fuzzy modeling; integrates uncertainty directly into ranking via belief-based distance; allows customization of utility functions and linguistic scales to context; combines performance level and stability when used with entropy.	Requires defining appropriate utility functions and fuzzy linguistic scales; slightly more computationally intensive than simple averages.
TA (Arithmetic Mean)	Simple and easy to compute; widely understood by practitioners and the public; requires minimal parameter choices.	Treats ordinal categories as equally spaced, which can distort meaning; ignores uncertainty in ratings; less sensitive to subtle differences between similar performers.
Entropy Analysis	Measures variability/stability directly; useful for detecting inconsistent or polarized feedback; independent of performance magnitude.	Does not provide direct performance ranking; high stability does not necessarily indicate high satisfaction; cannot replace satisfaction measurement, only complements it.

Source: Author’s elaboration.

## Data Availability

Data supporting reported results can be found in [35] (accessed on 12 July 2025).

## Abbreviations

The following abbreviations are used in this manuscript:

SERVQUAL	Service Quality
SERVPERF	Service Performance
MCDM	Multi-criteria decision-making
AHP	Analytic Hierarchy Process
TOPSIS	Technique for Order Preference by Similarity to Ideal Solution
VIKOR	VIšekriterijumska optimizacija i kompromisno rangiranje
MUSA	MUlticriteria Satisfaction Analysis
BS	Belief Structure
FBS	Fuzzy Belief Structure
ASI	Airline Satisfaction Index
BS-TOSIE	Belief Structure-Based TOPSIS for Survey Item Evaluation
PIBS	Positive Ideal Belief Solution
NIBS	Negative Ideal Belief Solution
